# Draft genome sequence of cellulose-degrading *Bacillus stercoris* BHUJPV-SS7 isolated from soil mixed with wood powder

**DOI:** 10.1128/mra.01072-23

**Published:** 2024-08-09

**Authors:** Saurabh Singh, Arthur Prudêncio de Araujo Pereira, Thierry A. Pellegrinetti, Jay Prakash Verma

**Affiliations:** 1 Plant-Microbe Interaction Lab, Institute of Environment and Sustainable Development, Banaras Hindu University, Varanasi, Uttar Pradesh, India; 2 Soil Science Department, Soil Microbiology Laboratory, Federal University of Ceará, Fortaleza, Ceará, Brazil; 3 University of São Paulo, Centre for Nuclear Energy in Agriculture, Piracicaba, São Paulo, Brazil; University of Strathclyde, Glasgow, United Kingdom

**Keywords:** genome sequence, *Bacillus stercoris*, cellulose-degrading

## Abstract

We report a complete genome of *Bacillus stercoris* BHUJPV-SS7 isolated from soil which contains 4,299 predicted genes and 4,012 predicted protein-coding genes within its chromosome (4,115,399 bp), and has 43.51% G + C content and a predicted beta-1,4-glucanase (EC 3.2.1.4) gene.

## ANNOUNCEMENT

*Bacillus* spp. commonly found in the rhizosphere soils (1) are known to harbor many beneficial properties including the ability of cellulose degradation (2). The bacteria described could be a potent cellulose degrader and hence whole-genome sequencing of *Bacillus stercoris* BHUJPV-SS7 was done.

*Bacillus stercoris* BHUJPV-SS7 was isolated from rhizosphere soil [collection depth 10 cm from surface (25.267270 N, 82.987285 E)], near a mango tree, recently fallen, and trunk degraded from inside leading to wood powder mixing with soil. The soil and pruned wood powder, collected simultaneously, were homogenized using a handheld homogenizer, serially diluted in 0.85% NaCl water, and spread after dilutions (from 10^−2^ to 10^−7^) on CMC (carboxymethyl cellulose) agar plates (0.5 g KH_2_PO_4_, 0.25 g MgSO_4_, 2 g CMC, 15 g agar, 0.2 g Congo-Red, and 2 g gelatin; 1,000 mL water at pH 6.8–7.2) and incubated for 5 days at 30°C. Subsequent subculturing (three times) in the same media produced multiple colonies with good zones (>15 mm clearance) of cellulose degradation and were selected for further assessment. Single colonies were cultured in nutrient agar (peptone 5 g/L, beef extract 3 g/L, agar 20 g/L, NaCI 0.5 g/L) media and incubated in a biological oxygen demand incubator for 72 h at 30°C. Enzyme quantification was performed on selected strains to select BHUJPV-SS7 for whole-genome sequencing.

Genomic DNA isolated using QIAGEN kit (cat. no: 51304) was checked using a Qubit 3 Fluorometer with dS DNA HS Dye (Thermo Fisher Scientific, Waltham, MA, USA). DNA samples were purified using AMPure beads, and enriched through PCR amplification (six cycles), using NEBNext Ultra II Q5 Master Mix (New England Biolabs), Illumina universal primer, and sample-specific octamer primers. Fragment analysis was done on Agilent 2100 Bioanalyzer with 1 µL library, sequenced on Illumina HiSeq 4000 (Illumina, Inc., San Diego, CA, USA).

The raw sequences were quality controlled using Fastqc v.0.11.9 (3) and Multiqc v.1.10.1 (4) and the sequence adapters were removed using Trimgalore v.0.6.6 (5). Default parameters were employed for all softwares in this study. The trimmed reads were assembled *de novo* using the Unicycler v.0.4.8 assembler (6). The final assembly was functionally annotated using RASTtk v.1.073 (7) and by NCBI Prokaryotic Genome Pipeline v.6.6 (8). Genome quality and taxonomic validation, including average nucleotide identity (ANI), were conducted using CheckM v.1.2.2 (9) and fastANI v.1.33 (10). The presence of beta-1,4-glucanase gene was predicted using dbCAN2 HMMs of CAZy v.1.9.1 families—v.10 in the KBase platform (11–13).

17,727,601 paired-reads of 151 bp were generated in this project. The draft genome measured 4,117,388 bp with 43.51% of average G + C content and median coverage of 1,110×. Genome assembly revealed 29 contigs with N50 value of 912.3 kilobases. The taxonomic annotation of the draft genome of strain BHUJPV-SS7 was most closely affiliated to *Bacillus stercoris* (ASM2055188v1) with 98.76% identical by ANI results. A total of 62 genes linked to carbohydrate degradation were predicted, including glycoside hydrolases, one gene for polyphenolic degradation, one for lignin degradation, and 11 lignin degradation auxiliary genes indicating potential for cellulose degradation and biomass biovalorization.

## Data Availability

The *Bacillus stercoris* BHUJPV-SS7 genome sequence has been deposited in DDBJ/EMBL/GenBank under accession number JALHBN000000000. Reads are available at the Sequence Read Archive (SRA) under accession number SRX14868609 and BioSample number SAMN26982665 (SRA accession number PRJNA820196). Genome annotations, including those generated by RAST and CAZy, are available at https://doi.org/10.5281/zenodo.7869037 .
